# The crystal structure of the chitinase ChiA74 of *Bacillus thuringiensis* has a multidomain assembly

**DOI:** 10.1038/s41598-019-39464-z

**Published:** 2019-02-22

**Authors:** Estefania O. Juárez-Hernández, Luz E. Casados-Vázquez, Luis G. Brieba, Alfredo Torres-Larios, Pedro Jimenez-Sandoval, José E. Barboza-Corona

**Affiliations:** 1 Universidad de Guanajuato Campus Irapuato-Salamanca, División de Ciencias de la Vida, Posgrado en Biociencias, Irapuato, Guanajuato, 36500 Mexico; 2 Universidad de Guanajuato Campus Irapuato-Salamanca, División de Ciencias de la Vida, Departamento de Alimentos, Irapuato, Guanajuato, 36500 Mexico; 3 Laboratorio Nacional de Genómica para la Biodiversidad, Centro de Investigación y de Estudios Avanzados del Instituto Politécnico Nacional (LANGEBIO-CINVESTAV), Apartado Postal 629, Irapuato, Guanajuato, 36824, Mexico; 4 Departamento de Bioquímica y Biología Estructural, Instituto de Fisiología Celular, Universidad Nacional Autónoma de México, Circuito Exterior s/n, Ciudad Universitaria, Apartado Postal 70–243, Ciudad de México, 04510 Mexico

## Abstract

There is no structural information about any chitinase synthesized by *Bacillus thuringiensis*, the most successful microbial insect larvicide used worldwide. In this study, we solved the 3D structure of the chitinase ChiA74 at 2.26 Å. The crystal structure shows that ChiA74 is composed of a modular arrangement formed by (i) a catalytic region (CD), (ii) a chitinase insertion domain (CID), (iii) a fibronectin type III domain (FnIII), and (iv) a chitin binding domain (CBD). The location of the CBD with respect to the CD has no structural similarity to other chitinases with known structures. The activity of a ChiA74 lacking its secretion signal peptide (ChiA74Δsp) and a truncated version lacking its CBD/FnIII domains (ChiA74Δsp-50) did not have statistical differences in activity against colloidal chitin. However, ChiA74Δsp exhibits 4.5 and 2.0 higher activity than versions lacking the CBD (ChiA74Δsp-60) and CBD/FnIII domains (ChiA74Δsp-50), respectively, when crystalline chitin was used as substrate. Our data suggest that the CBD might plays a significant role in crystalline chitin hydrolysis. We also demonstrated the importance of the catalytic E211 in the CD, as mutants ChiA74Δsp_E211N_ and ChiA74Δsp_D207N, E211N_ were inactive against colloidal and crystalline chitins, chitosan and 4-MU-GlcNAc_3_. ChiA74 has a processive activity producing oligosaccharides with degree of polymerization (DP) of 1 (GlcNAc) and 2 (GlcNAc_2_).

## Introduction

Chitinases are secreted by many prokaryotes and eukaryotes and are utilized for hydrolysis of chitin (a homopolymer of N-acetyl-D-glucosamine, GlcNAc), a rich ubiquitous nutrient resource^1^. According with the CAZy database (http://www.cazy.org/Glycoside-Hydrolases.html), chitinolytic enzymes are classified as chitinases (EC 3.2.1.14) and β-*N*-acetylhexosaminidases (EC 3.2.1.52), formerly called endochitinases and exochitinases, respectively. The first type of enzyme cleaves the chitin chain at internal sites randomly, whereas the second one hydrolyzes chitin from the nonreducing end of chitin by removing GlcNAc residues. Chitinases and β-*N*-acetylhexosaminidases are classified into four (18, 19, 23 and 48) and six (3, 5, 18, 20, 84, 116) glycoside hydrolases (GH) families, respectively, based on their amino acid sequence similarities^2^. Most bacterial chitinases belong to the GH 18 family (GH-18), and share a TIM-barrel fold in their catalytic subunit, have a conserved DxDxE motif, and employ a substrate-assisted catalytic mechanism^3^. Structural studies of different chitinases show that subsites +1 and +2 of the enzyme interacts with the reducing end of the chitin, and the hydrolysis occurs in the binding cleft between subsites −1 and +1^4^. Family 18 chitinases are classified into three subfamilies (A, B, C), based on the amino acid sequence similarity. Chitinases from subfamily A have a modular structure composed of the catalytic domain (CD), a fibronectin type III domain (FnIII), and a chitin binding domain (CBD). One of the main structural differences between the three subfamilies is that subfamily A has a small α + β domain (chitinase insertion domain, CID) inserted into the TIM barrel catalytic domain, which is absent in the subfamily B. Also, the substrate-binding cleft of chitinases from subfamily A is deeper than that observed in subfamilies B and C^5–10^. Although the definitive role of the CID has not been completely understood, it seems that its insertion into the TIM domain facilitate the orientation and strong association of the enzyme to the substrates^10^. CIDs have been reported in chitinases from *Vibrio harveyi*, the human cartilage glycoprotein-39, *Bacillus thuringiensis* and *Serratia marcescens*^7,11–13^. When the CID of *S. marcescens chiA* was deleted, the truncated enzyme showed a shallower tunnel in the catalytic domain than that of the intact enzyme, and also had reduction in thermal stability, specific activity and the substrate/product specificities^13^. A multiple sequence and structural alignment of twenty-seven CIDs shows the conservation of two motifs, YxR and [E/D]xx[V/I]. These conserved amino acids are predicted to be important for the interaction with the substrate through salt bridge and hydrogen bonds. Also five residues were identified into the CID that might be implicated in the formation of the hydrophobic core, as well as the stabilization of the domain^10^.

*B. thuringiensis* is the most successful microbial insect larvicide used worldwide. Its toxic effect is primarily due to Cry (crystal) and Cyt (cytotoxic) proteins that aggregate into stable crystalline inclusions during sporulation^14,15^. Although *B. thuringiensis* is most known for the plethora of Cry and Cyt proteins it produces, strains of this microbe produces other proteins that have applied biotechnological value, including chitinases, vegetative insecticidal proteins (Vip) and enhancing-like proteins, antimicrobial peptides and parasporins which can be used to synergize the activity of Cry proteins, to control lepidopteran and coleopteran pests, to inhibit food-borne pathogenic bacteria and to kill human cancer cells of various origins, respectively^1,14,16^. In particular, ChiA74, a chitinase synthesized by a Mexican strain of *B. thuringiensis* subsp. *kenyae*, has been shown to synergize the insecticidal activity of Cry proteins, and to generate chitin-oligosaccharides with antimicrobial activity^17–19^. This chitinase has a molecular mass of ~74 kDa, and its bioinformatic analysis predicts that it is composed of four domains: TIM-barrel, chitinase insertion (CID), fibronectin type III (FnIII) and chitin binding domains (CBD)^12^. The presence of a putative CID in a chitinase of *B. thuringiensis* was first reported by Juárez-Hernández *et al*.^12,20,21^. It has been shown that when ChiA74 is secreted, a protein of ~70 kDa and two truncated versions of ~60 and 50 kDa, which are processed from the C-terminal end are generated by an unknown mechanism^12,22^. Biochemical data demonstrated that the 50 kDa containing the (α/β)_8_-TIM-barrel plus the CID is the minimum version of the enzyme that possesses similar activity and catalytic efficiencies (kcat/Km) that ChiA74 lacking its secretion signal peptide (i.e. ChiA74Δsp) when colloidal chitin is used as substrate^12^.

To date, the three-dimensional (3D) structures for several proteins of biotechnological interest synthesized by *B. thuringiensis* have been elucidated^15,23–27^. However, there is no report about the tertiary structure of a chitinase synthesized by this bacterium. In this study, we solved the 3D structure of chitinase ChiA74 and demonstrated the importance of the conserved motif in the catalytic domain by site-directed mutagenesis. Our data demonstrate that this enzyme has a modular arrangement formed by four domains: (i) the catalytic region with an (α/β)_8_-TIM-barrel as a core structure, (ii) the chitinase insertion domain as a barrel insertion, (iii) the fibronectin type III and (iv) a chitin binding domain.

## Results

### Overall structure of ChiA74

Recombinant ChiA74 contains 676 amino acids including a N-terminal His-tag. ChiA74 crystallized in space group P 1 21 1 (Table 1). The cell content analysis in CCP4 suggest two copies of the molecule in the asymmetric unit. A molecular replacement solution was found with Phaser-MR using an alanine truncated model of the chitinase A1 from *B. circulans* (PDB ID 1ITX) as the search model. Side chains were completed from the initial MR solution in Coot as supported by electron density. The rest of the model was built manually in Coot (including the FnIII and the CBD) and several cycles of refinement in REFMAC5 were performed. The structure was refined to 2.26 Å resolution with a final *R*_*work*_ and *R*_*free*_ of 23.9% and 25.3%, respectively (Table 1**)**. No electron density was observed for the N-terminal His-tag, residues 572 to 581 and residues 675 to 676, and were not included in the final model. Residues from the CBD showed poor electron density that may indicate its high flexibility. However, most of the CBD was modeled as alanines due to the poor electron density maps.

**Table 1:** Data collection and refinement statistics of ChiA74.

PDB code	6BT9
*Data collection*	
Space group	P 1 21 1
Cell dimensions	
*a, b, c* (Å)	85.8 82.4 101.6
*α, β, γ* (°)	90, 108.1, 90
Resolution (Å)	62.76-2.26 (2.31–2.26)*
*R*_merge_	0.155 (0.712)
Mean I/σ(I)	8.9 (1.9)
CC_1/2_	0.965 (0.847)
Completeness (%)	99.7 (99.1)
Redundancy	5.4 (4.9)
*Refinement*	
Resolution (Å)	62.76-2.26
No. reflections	63155
*R*_work_/*R*_free_	23.9%/25.3%
No. non-hydrogen atoms	9450
Protein	9370
Water	78
B-factors	
Protein	24.32
Ligands	15.46
Water	11.79
Root mean squared deviations	
Bond lengths (Å)	0.0103
Bond angles (°)	1.354

^*^Values in parentheses are for the highest resolution shell.

**Figure 1:**
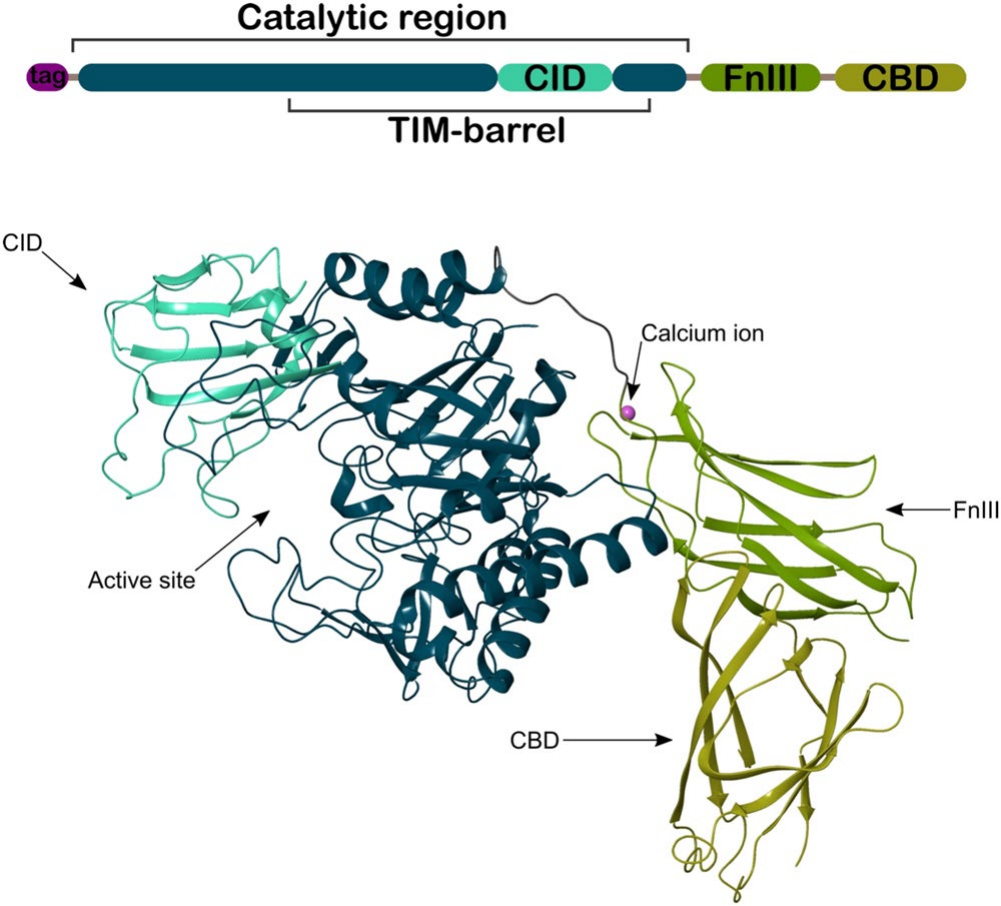
Overall structure of ChiA74. The scheme of the multidomain enzyme is shown at the top of the image. The catalytic region is composed of the characteristic (α/β)_8_-TIM-barrel and the chitinase insertion domain (CID), in dark blue and cyan, respectively. The fibronectin type III (FnIII) and the chitin binding domain (CBD), in green and lemon colors, respectively, are located at the C-terminal region. The rest of the image shows the active site into the cavity formed between the CID and the (α/β)_8_-TIM-barrel, indicated with an arrow. Two loops of the FnIII domain (green) and the linker loop to the (α/β)_8_-TIM-barrel keeps a highly coordinated calcium ion. The CBD at the C-terminal is shows in lemon color. The image only shows one molecule, for clarity, of two contained in the asymmetric unit.

The crystal structure of the ChiA74 consists of an (α/β)_8_-TIM-barrel, a CID, a FnIII and a C-terminal CBD (Fig. 1). Chitinases from family 18 share a common active site shape, which is often called “tunnel shaped”^28^. The catalytic region is composed of 442 residues and forms a substrate binding cleft and a semi-closed tunnel-shaped active site, with both the (α/β)_8_-TIM-barrel and the CID, similar to other chitinases with CID, the substrate binding groove is partially closed^29^. Three disulfide bonds between residues C78-C100, C140-C146, and C453-C461 are located in the TIM-barrel, with an additional one formed between residues C348-C359 in the CID domain. Structural comparison of the catalityc region of ChiA74 with other chitinases shows structural differences (Fig. S1) Although the catalytic region of chitinase A1 of *B. circulans* WL-12 shows high similarity with ChiA74 (∼60% of identity), ChiA74 differs mainly in a loop (residues 454 to 465) that is close to the CID (Fig. S1, loop in magenta) and in two segments of the CID-loop 1 defined as l′ and l″ (Fig. S1, regions of the CID-loop 1 are indicated in yellow and red, and corresponds to residue range 361 to 366 and 375 to 380, respectively). The two regions of the CID-loop 1 (l′ and l″), the loops of the TIM-barrel harboring the CID (Fig. S1, loops showed in magenta and orange), and the TIM-barrel loop 2 (Fig. S1, showed in green) are the main structural differences of ChiA74 with chitinase A from *S. marcescens*, chitinase A from *V. harveyi* and ChiW from *Paenibacillus sp*. str. FPU-7 (Fig. S2, PDB code 1FFR, 3B9A and 5GZT, respectively). ChiW from *Paenibacillus sp*. str. FPU-7 differs even more on its TIM-barrel loop 2, which is considerable smaller to the rest of the chitinases showed in Fig. S1.

In our structure, the FnIII-like domain is linked to the TIM-barrel through residues 476 to 484 at the opposite site of the catalytic region, defined as FnIII linker loop. A calcium ion is highly coordinated by residues D483 and E485 (witin the FnIII linker loop), residues D512 and N513 (within the FnIII loop 2 which harbors residues 508 to 516) and D554 (within the FnIII loop 6, residues 554 to 562); these loops are the linkers between β strands of the domain, i. e. loop 2 links β strand 2 to 3, and loop 6 links β strand 6 to 7. Other contacts of the N-terminal region of the FnIII occur with residues E248 to K251 of the TIM-barrel. FnIII does not form an extended substrate binding surface with the catalytic cleft as has been observed in other chitinases, for instance, chitinase ChiA of *Serratia marcescens*, and ChiA of *V. harveyi*, where this domain has multiple sites for substrate binding along the surface towards the active site, and which functions in enhancing binding and hydrolyzing activities against natural chitin^30,31^. The crystal structure of ChiA74 and the catalytic parameters of the truncated FnIII version of the enzyme^12^, shows that FnIII (i) lacks exposed aromatic residues, and (ii) acts as a stable linker between the catalytic region and the CBD. A similar role of a FnIII domain has been suggested in chitinase ChiW from *Paenibacillus* sp. str. FPU-7^32^, and other multimodular carbohydrate-interacting proteins containing FnIII domains^33^. Additionally, CBD shares interfaces between both FnIII and the (α/β)_8_-TIM-barrel. Briefly, N664 to E667 form hydrophobic and electrostatic interactions with the β-sheet 7of the FnIII (residues 563 to 568), and its loop 1 interacts with the α -helix 4 of the TIM-barrel (Figs 1 and S2).

### Structure of the CID of ChiA74

**Figure 2:**
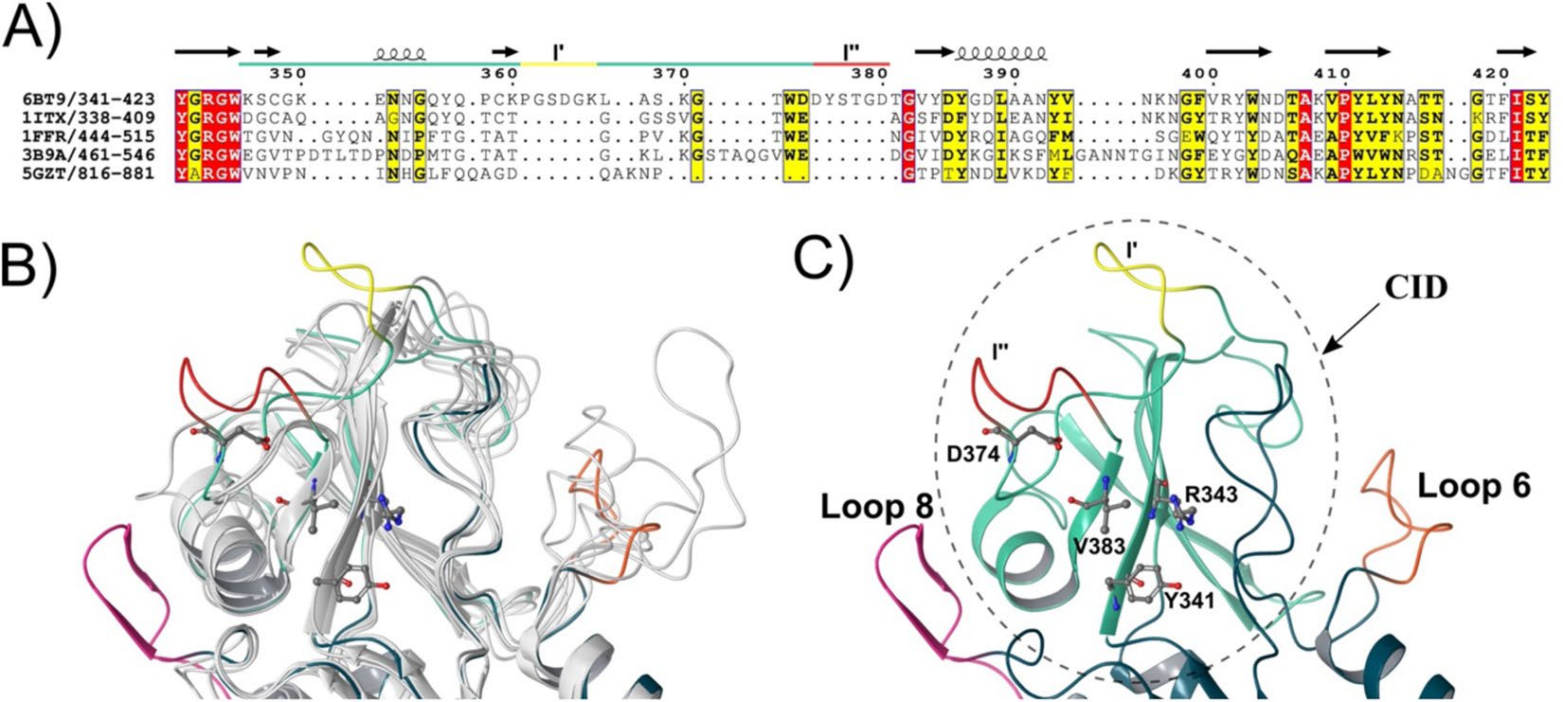
Structure-based sequence alignment of ChiA74 with other chitinases. The image shows the structure-based sequence alignment of the catalytic cleft of ChiA74 and others CID containing chitinases. Similar and conserved residues are indicated in yellow and red, respectively, according with ENDScript server^35^. UCSF Chimera^34^ for structures superimposing. (**A**) Structure-based sequence alignment, only the region of the CID is shown. Main difference with other CIDs is the CID-loop 1 long. Regions l′ and 1″ are indicated in yellow and red, respectively. The PDB ID are the following: 6BT9 (chitinase ChiA74 from *B. thuringiensis*); 1ITX (catalytic domain of chitinase A1 from *B. circulans* WL-12); 1FFR (chitinase of mutant Y390F complexed with hexa-N-acetylchitohexaose from *S. marcescens*); 3B9A (*V. harveyi* chitinase A complexed with hexasaccharide) and 5GZT (chitinase ChiW from *Paenibacillus* sp. str. FPU-7). (**B**) Structural superimposition of ChiA74 with other chitinases where the differences between structures are shown with color. PDB codes of the superimposed structures are the same used in A. (**C**) CID in ChiA74 (dashed line) protrudes from Loops 6 and 8 of the TIM-barrel. Residues that would be interacting with substrate are indicated. Region 1′ of the CID-loop1 closes the substrate binding groove ″ of the active cleft (yellow).

The CID is located between the seventh alpha-helix and the seventh beta-strand of the (α/β)_8_-TIM-barrel. Residues in this loop assemble the “half active site wall” of the entire catalytic region. The CID consists of 82 residues, constrained between residues 341 and 423. In ChiA74 this domain is composed by five antiparallel beta-strands, one α -helix and one loop of 38 residues long between its first and the second β-strand of the CID, defined as CID-loop 1. A structure-based sequence alignment and a structural comparison with other chitinases (Fig. 2**)** carried out by the structure comparison tool MatchMaker of the UCSF Chimera program^34^ showed that the CID-loop 1 is longer in ChiA74, with two segments that extend and closes the catalytic cleft, named as l′ and l″. CID-loop 1 is the less conserved region compared with similar chitinases of known structure. PDB codes indicated in Fig. 2A, and similar, conserved residues are indicated in yellow and red, respectively, according with ENDScript^35^. Those segments are considerably long when compared with other structures (Fig. 2B). PDB codes of the superimposed structures are the same that are indicated in Fig. 2A, ribbon representation images were rendered in Maestro program^36^. The CID-loop 1 together with the TIM-barrel loop 2 (residues 74 to 147) (Fig. S1) assemble into a structure that partially closes the substrate binding groove. This assembling is characteristic of exo-acting processive enzymes during the degradation of non-soluble polysaccharides^37^, since processive chitinases possess partially closed tunnels at the active site and conserved aromatic residues that bind the substrate and allows substrate translocation and processivity^38^, and is also an important structural element for endo-acting chitinases on soluble substrates^28,32^. Additionally, four residues in the CID are suggested to interact with the substrate at the catalytic cleft^10^; in ChiA74 these residues are Y341, R343, V383 and D374 (Fig. 2C).

### Structural details of the catalytic cleft of ChiA74

**Figure 3:**
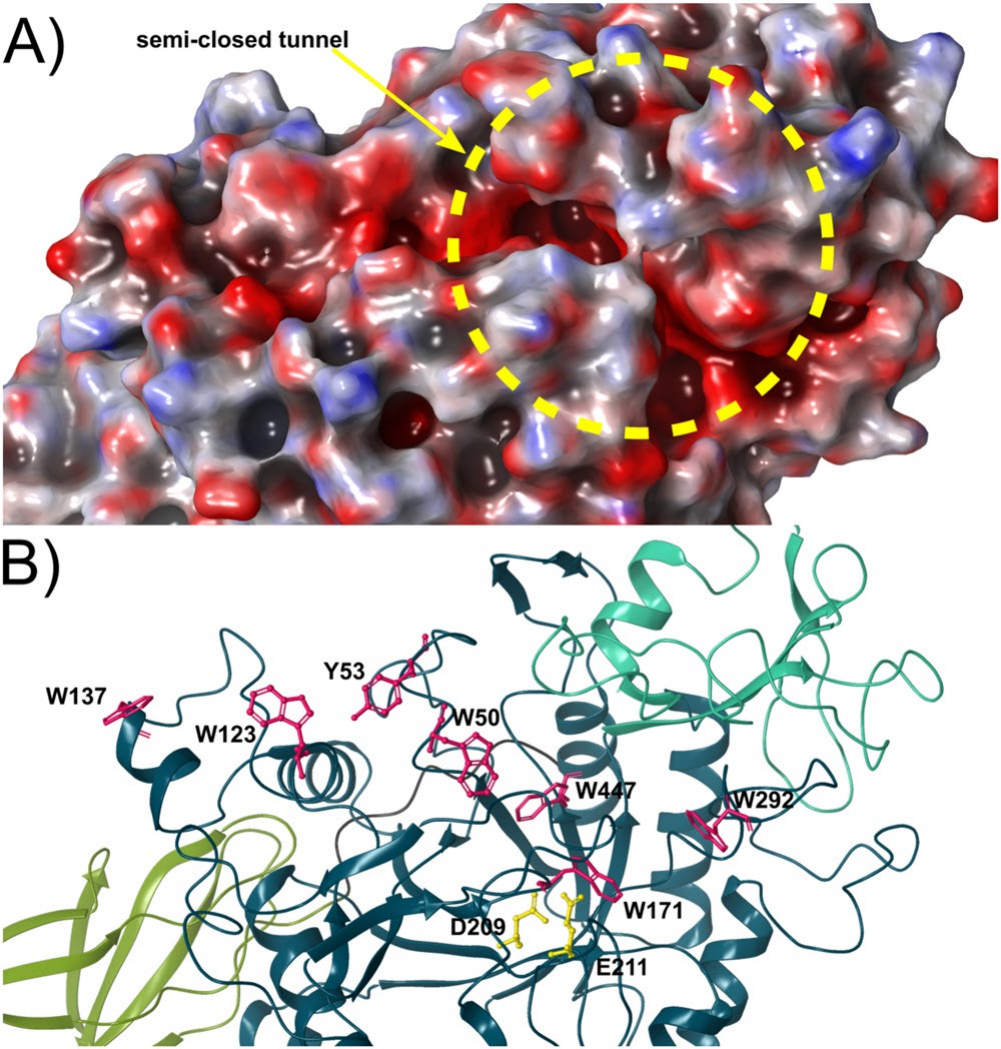
Structural details of the catalytic cleft in ChiA74. (**A**) Electrostatic surface potential of the semiclosed tunnel active site of ChiA74. Red and blue color indicate negative and positive charges, respectively, generated using Poisson-Boltzmann (APBS) method for the solvated molecule. The binding and catalytic area displays an extended negatively charged surface. The discontinued circle in yellow is indicating the semi-closed tunel. (**B**) A series of aromatic residues for substrate stabilization and binding are shown in magenta. Residues W137, W123, Y53, W50 and W447 form an extended surface towards the catalytic site (only D209 and E211 are shown, in yellow). Images are superimposables.

The catalytic semi-closed tunnel of ChiA74 formed by the TIM-barrel and CID displays a negatively charged surface (Fig. 3A). The (α/β)_8_-TIM-barrel harbors a series of aromatic residues through its molecular surface, that control substrate binding near the active site, subsites −5 to +2. The subsites −1 and +1 are between the cut-off site of the enzyme, the positive subsites (e.g.+2) toward the reducing end, and the negative subsites (−2, −3, −4, −5…) in the direction to the non-reducing end (Figs S3, S4B). Several studies in model enzymes from subfamily A of the family 18 chitinases^30,39^ indicate that conserved exposed aromatic residues at the entrance of the catalytic cleft play a key role in hydrolyzing the chitin chain, when using crystalline chitin as a substrate, by guiding the entrance of the substrate into the active site. In ChiA74, the aromatic residues at the entrance of the catalytic cleft correspond to W123 and W137 which are similarly located as in chitinase from *B. circulans, S. marcescens* and *V. harveyi*, and may play the same role as substrate guiding residues (Figs 3B, S3). Residues Y53 and W50 are also conserved and they may be involved in extending from −5 to −3. These amino acids are structurally superimposable to Y56 and W53 of ChiA1 from *B. circulans*, and appear to be essential for crystalline chitin hydrolysis^30,40^. Due to their structural resemblance, residues Y53 and W50 may have the same function in hydrolyzing crystalline chitin. Residues W137, W123, Y53, and W50 are located on the extended surface at the entrance to the catalytic cleft in ChiA74, suggesting that this chitinase works as processive chitinases towards the non-reducing end of chitin^41^ predominantly releasing disaccharides and also monosaccharides at +1 and +2 subsites, as it was recently observed with purified ChiA74^22^. For ChiA74, residues stacking in subsites +1 and +2 are W171 and W292, respectively (Fig. S3).

The catalytic domain harbors the motif (DxDxE) which is conserved in most family 18 chitinases, and whose role in the catalytic process has been demonstrated in other bacterial chitinases^21,34–43^. In the current accepted model, the second Asp within the DxDxE motif turns towards the Glu after substrate binding, enabling hydrogen bond interactions with the substrate and Glu, then the glycosidic oxygen acts as a nucleophile and the second Asp deprotonates the N-acetamido nitrogen during oxazolinium ion formation. Finally the Glu protonates the glycosidic oxygen and deprotonates the nucleophilic water, giving the hydrolysis of the oxazolinium ion^44^. In ChiA74 this motif harbors the following amino acids: D207, D209, and the catalytic E211^21^ (Figs 3B, S3).

### Chitinase activity of truncated versions and catalytic motif mutants against different substrates

ChiA74Δsp and its versions lacking the chitin binding domain (ChiA74Δsp-60) and chitin binding-fibronectin type III domains (ChiA74Δsp-50) were assayed against three natural substrates (crystalline chitin, colloidal chitin, chitosan) and one synthetic compound, i.e. 4-MU-(GlcNAc)_3_ (Table 2). Also, mutants of ChiA74Δsp with one (ChiA74Δsp_E211N_) and two (ChiA74Δsp_mD207N, E211N_) amino acid replacements in the catalytic motif were generated, substituting D207 and the catalytic E211 (negative amino acids) with asparagines (N, uncharged amino acid). The importance of those amino acids in catalysis has been demonstrated in chitinase A1 of *B. circulans* and *S. marcescens*, primarily the glutamic acid which is directly involved in the hydrolysis of the oxazolinium ion intermediate^42,44^. In general, the highest activity with natural substrates was observed with colloidal chitin rather than crystalline chitin and chitosan in a time reaction of 12 h at 37 °C. As was previously reported^12^, we confirmed that ChiA74Δsp and ChiA74Δsp-50 show the highest activity with colloidal chitin, without statistical difference. With crystalline chitin, ChiA74Δsp showed 4.5 and 2.0 higher activity than ChiA74Δsp-60 and ChiA74Δsp-50, respectively, but with chitosan the highest activity was detected with ChiA74Δsp-50. Using 4-MU-(GlcNAc)_3_, truncated versions showed differences in the activity against this substrate. When mutants (ChiA74Δsp_E211N_) and ChiA74Δsp_D207N, E211N_ were assayed with crystalline chitin, colloidal chitin, chitosan and 4-MU-(GlcNAc)_3_, no enzymatic activity was observed, demonstrating the importance of the catalytic E211 in the chitinase activity^42,44^ of ChiA74Δsp.

**Table 2:** Chitinase activity (U/μmol) of different versions of ChiA74 against several substrates.

Enzymes	crystalline-chitin*	Colloidal chitin*	Chitosan*	4-MU-GlcNAc_3_**
ChiA74Δsp	97.46 ± 6^a***^	187.15 ± 13^a^	48.73 ± 23^a^	13093.16 ± 635^a^
ChiA74Δsp-60	21.54 ± 1^b^	60.53 ± 4^b^	28.52 ± 14^a^	10313.68 ± 500^b^
ChiA74Δsp-50	47.76 ± 3^c^	170.44 ± 12^a^	51.28 ± 25^a^	8846.54 ± 429^c^
ChiA74Δsp_E211N_	0.71 ± 0.0^c^	0.71 ± 0.05^c^	1.41 ± 0.7^b^	64.97 ± 3^d^
ChiA74Δsp_D207N, E211N_	1.77 ± 0.1^d^	3.53 ± 0.25^d^	2.82 ± 1^b^	51.55 ± 2^e^

*One Unit of chitinase activity was defined as the amount of enzyme required to release 1 µmol of NAG in 1 h.

**One Unit of chitinase activity was defined as the amount of enzyme required to release 1 µmol of MU in 1 h.

***Different letter in the same column (a, b, c) indicate significant difference at P < 0.05 determined by the Tukey multiple range test.

On the other hand, TLC (Fig. S4) was carried out with colloidal chitin and crystalline chitin, the natural substrates that were most susceptible to the hydrolytic activity of purified ChiA74Δsp and ChiA74Δsp-50. Chitin-oligosaccharide derivatives with degree of polymerization of 1 and 2 were detected, as previously reported^22^, which suggest a processive activity in substrate degradation. Similar results were reported with BthChi74, a chitinase that shares 98% identity with ChiA74^45^.

## Discussion

The crystal structure of ChiA74 shows that this enzyme has a modular structure formed by four domains: (i) the catalytic region with an (α/β)8-TIM-barrel as a core structure, (ii) the chitinase insertion domain as a barrel insertion, (iii) the fibronectin type III and (iv) a chitin binding domain, as previously hypothesized by Juárez-Hernández *et al*.^12^.

The modular organization of ChiA74 has similarities, but it is not identical to the structure reported for other bacterial chitinases (e.g. ChiA1 from *B. circulans*, ChiA from *S. marcescens*, ChiA from *V. harveyi*, ChiW from *Paenibacillus* sp. str. FPU-7^30-32,40^. Some chitinases have immunoglobulin-like fold domains (FnIII, CBD or both), but ChiA74 differ from those enzymes mainly at the structural arrangement level, because of the very closed substrate binding groove of the catalytic cleft and in the multimodular assembly (Figs 1, 2). The TIM-barrel/CID catalytic domain of ChiA74 harbors the conserved motif in most family 18 chitinase involved in chitin hydrolysis (i.e. DxDxE), where E211 plays an important role as the catalytic amino acid initiator of the oxazolinium ion intermediate hydrolysis^42,44^. We demonstrated the importance of residues present in the conserved motif of the catalytic domain by site-directed mutagenesis, in particular, the significance of the catalytic E211 in ChiA74, as mutants ChiA74Δsp_E211N_, ChiA74Δsp_D207N, E211N_ were inactive against two natural polysaccharides (i.e. chitin, chitosan) and one short synthetic substrate (4-MU-GlcNAc_3_) (Table 2), similar as reported for other chitinases^42^. The catalytic domain of ChiA74 from *B. thuringiensis* shows high identity with ChiA of *S. marcescens*, suggesting that ChiA74 might has similar catalytic mechanism. In the proposed mechanism for ChiA74, D209 interacts with D207, after binding of substrate D209 flips to its alternative position where it interacts with E211 forcing the substrate acetamido group of −1 sugar to rotate around the C2-N2 bond (Fig. S5)^29^.

As ChiA74 contains a CID inserted into the TIM barrel catalytic domain, this enzyme can be classified in the subfamily A of the family 18 chitinases^10^. The location of the FnIII and CBD in ChiA74 is different with respect to other chitinases of the family 18, in which the FnIII domain is often located along the catalytic region and functions as a chitin binding module^29,46^. In ChiA74, the FnIII might act as a stable linker between the catalytic regions and the CBD in a similar way as has been proposed in ChiAW fom *Paenibacillus* sp. str. FPU-7 and *Microbacterium aurum* B8^32,33^. The modular arrangement of ChiA74 represents the first report of a full version chitinase structure with a CBD located slightly distal from the catalytic region linked by a FnIII domain, and suggests a different role (substrate binding and guiding to the active site) for the CBD. The location of the CBD with respect to the catalytic region has no structural similarity with other chitinases of known structure, and to date, represents the first report of this particular multimodular assembly. The structural arrangement of this domain remains unclear, but our data indicates that this domain could be important for crystalline chitin hydrolysis. For example, the activity of the truncated version against crystalline chitin is lower (from 0.22 X to 0.49X) than ChiA74Δsp, suggesting that CBD is necessary for crystalline chitin hydrolysis. We do not have experimental evidence, but as crystalline chitin has a fibrillar structure with high degree of crystallinity and polymorphism, and the chitin binding domain surface might facilitate the movement of the chitin chain toward the catalytic region, as suggested previously^7^ or prevent dissociation from the crystalline substrate. In general, we observed a higher chitinase activity when using colloidal chitin as a substrate than using chitin in its crystalline form. It is likely that colloidal chitin might interact more easily with the enzyme because the crystalline structure maintained by hydrogen bonds is disrupted due to the acid treatment, thus facilitating the interaction of the sugar chains of the colloidal chitin with ChiA74. Similar results have been observed with a chitinase from *V. harveyi*^7^. Our data confirmed that ChiA74Δsp and the truncated version ChiA74Δsp-50 (i.e., the enzyme version with the CD and CID) have the same activity against colloidal chitin, indicating the CBD and FnIII are not necessary for the chitinase activity against this substrate^12^. When crystalline and colloidal chitin were treated with ChiA74Δsp and analyzed by TLC, oligosaccharides of DP of 1 (GlcNAc) and DP of two (GlcNAc_2_) were obtained, indicating that ChiA74Δsp has an exo-acting processive activity which is congruent with the presence of a partially closed substrate binding groove formed by the CID-loop 1 and the TIM-barrel loop 2, characteristics of enzymes with this activity^37^. Similar results were obtained previously in our laboratory using purified ChiA74Δsp and colloidal chitin^22^. The crystal structure and TLC data suggest that ChiA74 probably hydrolyses chitin chains from reducing toward non-reducing end, similar to ChiA by *S*. *marcescens*^47^. We previously reported that ChiA74 has an endochitinase activity which was determined using crude extracts and synthetic fluorescent substrates^21^. However, the putative endochitinase activity reported in that work might be erroneous considering the fact that ChiA74 was mixed with other proteins in the crude extract and the substrate was not a chitin polymer. In this regard, in future studies it is very important to confirm or discard, experimentally, whether ChiA74 cleaves the chitin chain at internal sites or hydrolyzes chitin from the end in a processive mode, or both, using more sensitive techniques than TLC (e.g. HPLC)^44,47^.

Finally, we report for the first time the three-dimensional structure of a chitinase synthesized by *B. thuringiensis*, the most important microbial insecticide worldwide. The elucidation of the crystal structure of ChiA74 is an important advancement in the study of metabolites of *B. thuringiensis* and provides a foundation for future studies of chitinases synthesized by this bacterium, including protein engineering or directed evolution to enhance the applied versatility of these enzymes.

## Materials and Methods

### Bacterial strains and culture conditions

The recombinant plasmid, *pColdI-chiA74Δsp*, harbors *chiA74* lacking sequences coding for the secretion signal peptide, and its N-terminal end is fused to a heterologous sequence of 28 amino acids that includes the 6x-histidine tag and Factor Xa cleavage sites^12,22^. *Escherichia coli* TOP10 (Invitrogen, Carlsbad CA, EE.UU.) and *E. coli* BL21 Rosetta 2 (Merck Millipore, MA) were used as host cells for propagation or protein production, respectively. All bacteria were cultured at 37 °C in Luria-Bertani (LB) liquid medium (Invitrogen, Carlsbad CA, USA), supplemented with ampicillin (100 mg/ml) or chloramphenicol (34 mg/ml).

### Expression and purification of ChiA74Δsp

*E. coli* BL21 Rosetta 2/*pColdI-chiA74Δsp* was grown overnight in 10 ml of LB broth supplemented with ampicillin and chloramphenicol, and then transferred to 1000 ml fresh medium supplemented with the same antibiotics. Culture was grown at 37 °C/200 rpm to reach an OD_600_ of 0.5, and then incubated at 4 °C for 15 min. Bacterial culture was incubated with agitation at 15 °C for 30 min, and IPTG was added to a final concentration of 0.5 mM. The culture was incubated for 24 h at 16 °C, 200 rpm, and then centrifuged, the supernatant was discarded, and the pellet was resuspended and incubated on ice for 30 min in 25 ml of buffer A (100 mM Tris-HCl, pH 7; 500 mM NaCl, 10 mM imidazole) supplemented with lysozyme (1 mg/ml). Samples were sonicated 10x, 30 s each, at an amplitude of 30 Hz using a 20 kHz ultrasonic processor (Sonic and Materials, Inc., New-town, CT 06470-1614 USA). The extract was centrifuged 30 min at 13,000 *g* and the supernatant was passed through a HisTrap HP column (GE Healthcare Bio-Sciences AB, Upsala Sweden) pre-equilibrated with buffer A. Unbound protein was removed with 15 ml of buffer A, 15 ml of buffer A-20 mM imidazole, 15 ml of buffer A-40 mM imidazole, and finally samples were eluted with 3 ml of buffer A-500 mM imidazole. Eluted samples were dialyzed at 4 °C in buffer E (100 mM Tris-HCl, pH 7; 150 mM NaCl) without imidazole. Collected fractions were analyzed by 10% SDS–polyacrylamide gel (SDS–PAGE) and zymogram. Samples, purified with the HisTrap HP column were loaded onto a Superdex 200 10/300 GL (GE Healthcare life science) column previously equilibrated with buffer A (100 mM Tris–HCl pH 7.0, 25 mM NaCl) and ChiA74Δsp was separated by fast protein liquid chromatography (FPLC) (Biologic Duo-Flow Pathfinder 20 System BioRad, Hercules CA, USA). Fractions of 1 ml were collected at a rate of 0.5 ml/min using buffer A and monitored at 280 nm. Fractions in the peak were collected and analyzed by 10% SDS–PAGE and zymograms. Protein concentration was determined at 595 nm using the Quick Start Bradford 1 x Dye reagent (BioRad, HerculesCA, USA) in a Synergy HTX Biotek (Winooski, VT).

### SDS-PAGE and zymograms

Fractions containing ChiA74Δsp were monitored by SDS-PAGE and zymogram to corroborate the presence and activity of the purified recombinant protein. Portions of each fraction were treated with Laemmli’s disruption buffer supplemented with β-mercaptoethanol. Identical samples were fractionated by electrophoresis in a 10% SDS–PAGE. One gel was stained with coomassie blue and the second was washed with casein-EDTA buffer [1% (w/v) casein, 2 mM EDTA, 40 mM Tris–HCl, pH 9], and activity detected with 25 μM of the fluorogenic substrate 4-MU-(GlcNAc)_3_ (Sigma, St. Louis, MO), as previously described^21^.

### Protein crystallization and data collection

Initial crystallization tests were performed by using a Crystal Gryphon robot (Art Robbins Instruments, Sunnyvale CA, USA) using MRC 3 well crystallization plates (Jena Bioscience, Jena, Germany). Approximately 300 conditions were tested for crystallization at 292 K and crystals were formed in 30 conditions after two weeks using protein at 15 mg/ml, whereby a selection of the most appropriate crystals was made to improve their size. New drops were set in VDX plates (Hampton Research, Aliso Viejo CA, USA) using hanging-drop vapor diffusion technique by mixing equal volumes of the pure protein with selected precipitants from a sparse matrix screen. All the crystals were grown as a sheet clusters; no individual crystals were obtained even after crystal seeding. Crystals were selected for diffraction from a condition containing 25% (w/v) PEG 6000, 100 mM HEPES/NaOH pH 7.0 and 200 mM NaCl. A single sheet crystal was obtained by breakage and cryoprotected in a similar solution containing 20% (w/v) of glycerol prior to flash cooling in liquid nitrogen. Two crystals were used to collect diffraction data at 100 K using a MarMosaic 225 CCD detector at APS beam line LS-CAT 21-ID-F with 1° rotation per image.

### Structure determination and refinement

Diffraction intensities were integrated and scaled as monoclinic P 1 21 1 lattice symmetry with DIALS using xia2^48^ and AIMLESS^49^ from the CCP4 software suite, respectively. The program MATTHEWS_COEF^50^, through the cell content analysis task in CCP4 suggests two copies of ChiA74 in the asymmetric unit. A molecular replacement solution was found with Phaser-MR^51^, using a poly alanine model of the chitinase A1 structure from *B. circulans* (PDB ID 1ITX) as the search template. Residues of the catalytic region were modeled in Coot according with sequence as they were supported by electron density. Crystallographic refinement was performed with REFMAC5^52^. A total of 3145 reflections (5.0%) were used as the test set. Simulated annealing, individual atomic coordinate and individual atom isotropic displacement parameter refinement strategies were performed with non-crystallographic symmetry (NCS) restraints. The model was adjusted to improve the fit to likelihood weighted electron density maps using Coot^53^, and the rest of the model corresponding to the fibronectin type III and carbohydrate binding domains were constructed manually in agreement with electron density maps, in Coot. Some solvent molecules were added where they were supported by both chemistry and geometry. The quality and stereochemistry of the model were evaluated using Coot validation tools, Molprobity and the wwPDB Validation Server^54^.

### Construction of ChiA74 mutant

Mutations in the catalytic domain were carried out by site-directed mutagenesis using pCold-*chiA74*Δsp as template^22^. This recombinant plasmid was amplified with the Phusion high-fidelity DNA polymerase (Thermo Scientific, MA, USA) using 5′ phosphorylated oligonucleotides allowing the plasmid’s re-ligation. Two *chiA74*Δsp mutants, encoding proteins with one (E211N) or two (D207N, E211N) amino acids changes in the catalytic motif were obtained. The following primers were used, respectively: Fw E211N: 5′-CGTAGATTTAGACTGGAACTATCCGGGCGTTGAAA-3′, Rv E211N: 5′-TTTCAACGCCCGGATAGTTCCAGTCTAAATCTACG-3′, and Fw D207N,E211N: 5′-GATGGCGTAAACTTAGACTGGAACTATCCGGGCGTT-3′, Rv D207N,E211N: 5′-AACGCCCGGATAGTTCCAGTCTAAGTTTACGCCATC-3′. Amplicons were treated with DpnI to eliminate DNA templates and then visualized in 0.8% agarose. Samples were purified with the agarose gel extraction kit (Jena Biosciences, Jena, Germany) and dialyzed. *E. coli* Top10 was transformed with the purified amplicons by electroporation at 2.5 kV and transformants were selected on LB agar with ampicillin at 37 °C. The presence of point mutations was corroborated by sequencing and analyzed with the program LALING (https://embnet.vital-it.ch/software/LALIGN_form.html).

### Enzymatic activity

Chitinase activity was determined in triplicate assays at 37 °C in 20 mM phosphate buffer, pH 7, using the substrate 4-methylumbelliferyl β-D-N,N′,N′′-triacetylchitotrioside (4-MU-GlcNAc_3_) (Sigma, St. Louis, MO) and purified recombinant enzymes at final concentrations of 100 µg/ml. The amount of 4-MU released from the substrate was calculated spectrofluorometrically (excitation at 360 nm and emission at 455 nm) with a Synergy HTX Biotek (Winooski, VT) instrument using a 4-MU standard curve. One unit (U) of chitinolytic activity was defined as the amount of enzyme required to release 1 μmol of 4-methylumbelliferone in 1 h. To determine chitinase activity with crystalline chitin (Sigma-Aldrich, St. Louis MO, USA), colloidal chitin (Sigma-Aldrich, St. Louis MO, USA) and chitosan, a suspension containing 0.5% of these substrates was mixed with 0.036 mg/ml of purified enzyme to reach a final volume of 0.5 ml using 20 mM phosphate buffer, pH 7, and then incubated at 37 °C with gentle agitation. Aliquots of 40 μL were taken after 12 h of incubation and mixed with 40 μL of 3,5-dinitrosalicylic acid. The samples were boiled for 5 min, allowed to cool and water was added to a final volume of 200 μl, then the absorbance was measured at 540 nm to determine the concentration of reducing sugars^19,21^.

### Analysis of chitin hydrolysis with ChiA74Δsp using thin layer chromatography

Mixtures containing 0.036 mg/ml of ChiA74Δsp, 0.5% (w/v) colloidal chitin, and 20 mM phosphate buffer (pH 7.0) were incubated at 37 °C, 200 rpm for 12 h to generate chitin-derived oligosaccharides (OGS). Reaction was terminated by heating at 100 °C for 5 min. Samples were centrifuged, supernatants were collected and concentrated in a DNA120 SpeedVac (ThermoSavant), then pellets were resuspended in 10 µl of double distilled water and 10 µl of methanol. OGS were analyzed by silica gel (Merk) thin layer chromatography (TLC) using 5:4:3 (v/v/v) n-butanol: methanol:16% aqueous ammonia as the mobile phase. Samples were visualized by staining with 20% (v/v) sulfuric acid in ethanol and incubated at 80 °C until chitin-derived oligosaccharides signals were developed. Molecular markers (Sigma, St. Louis, MO) *N*-acetyl-D-glucosamine (GlcNAc) and *N,N*′*-*diacetylchitobiose (GlcNAc_2_) were used to determine the degree of polymerization (DP)^22^.

## Supplementary information

Supplementary figures

## Data Availability

All reagents and data described in this manuscript are available upon request. The coordinates and structure factors of ChiA74 have been deposited in the PDB under Accession code 6BT9.
