# Supplementary material for: The crystal structure of the chitinase ChiA74 of Bacillus thuringiensis has a multidomain assembly
Source: Sci Rep. 2019 Feb 22;9:2591. doi: 10.1038/s41598-019-39464-z (PMC6385353; doi:10.1038/s41598-019-39464-z)
Supplement: Supplementary file 1 — Supplementary figures [file 41598_2019_39464_MOESM1_ESM.pdf]

Supplementary figures

The crystal structure of the chitinase ChiA74 of *Bacillus thuringiensis* has a multidomain assembly

Estefania O. Juárez-Hernández, Luz E. Casados-Vázquez, Luis G. Brieba, Alfredo Torres-Larios, Pedro Jimenez-Sandoval and José E. Barboza-Corona

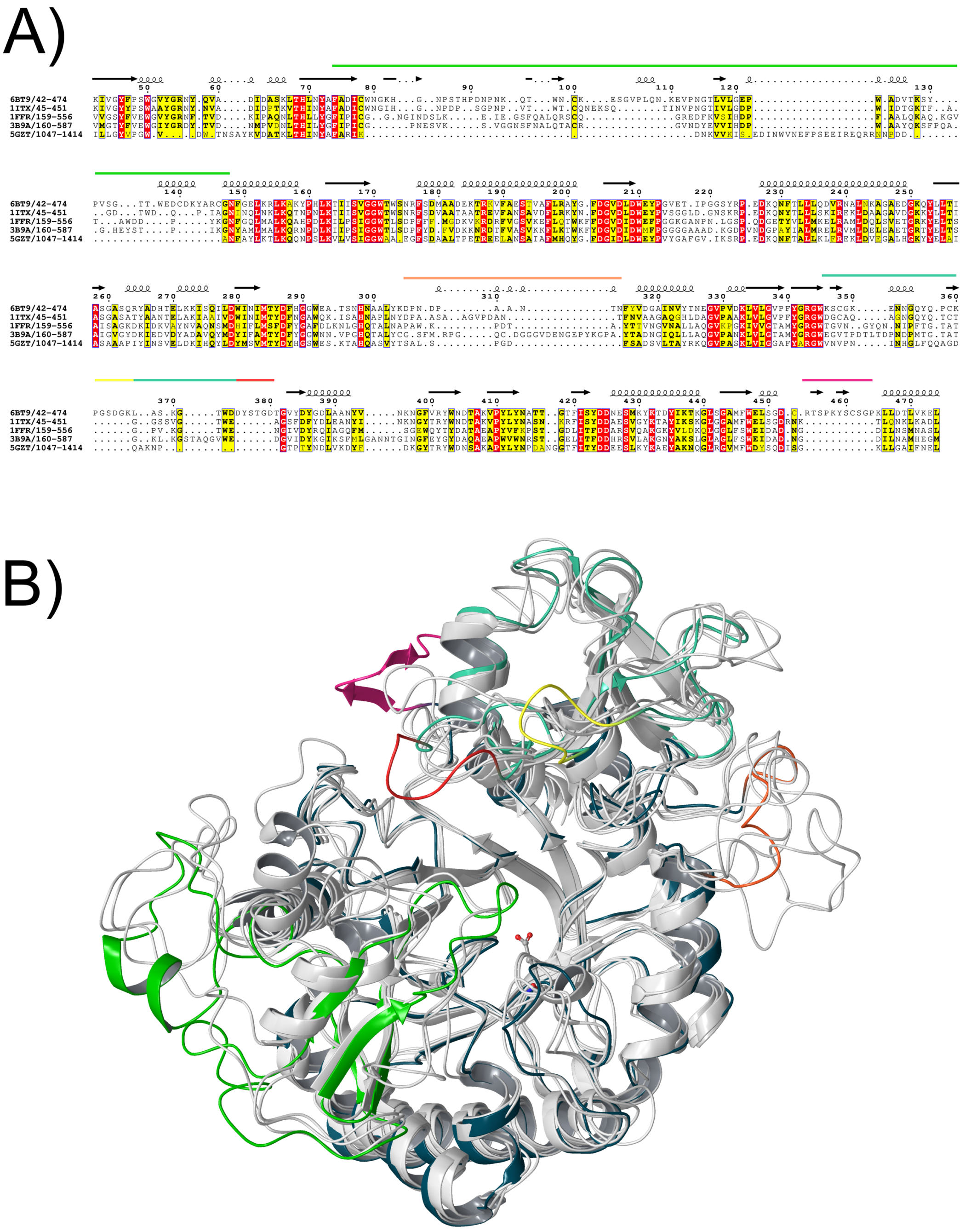

Figure S1. Structure-based multiple sequence alignment of the catalytic cleft. A) The image shows the structure-based sequence alignment of the catalytic cleft of ChiA74 and others CID containing chitinases. PDB codes and residue range are indicated in A and are 1ITX (residues 45-451), 1FFR (residues 159-556), 3B9A (residues 160-587), 5GZT (residues 1047-1414). Similar and conserved residues are indicated in yellow and red, respectively, according with ENDScript server. Analysis made on UCSF Chimera. B) Structural superimposition of ChiA74 with other chitinases. main differences are indicated in the sequence with the same color scheme. Catalytic glutamic acid is in the center of the image. Images were rendered in Maestro[36].

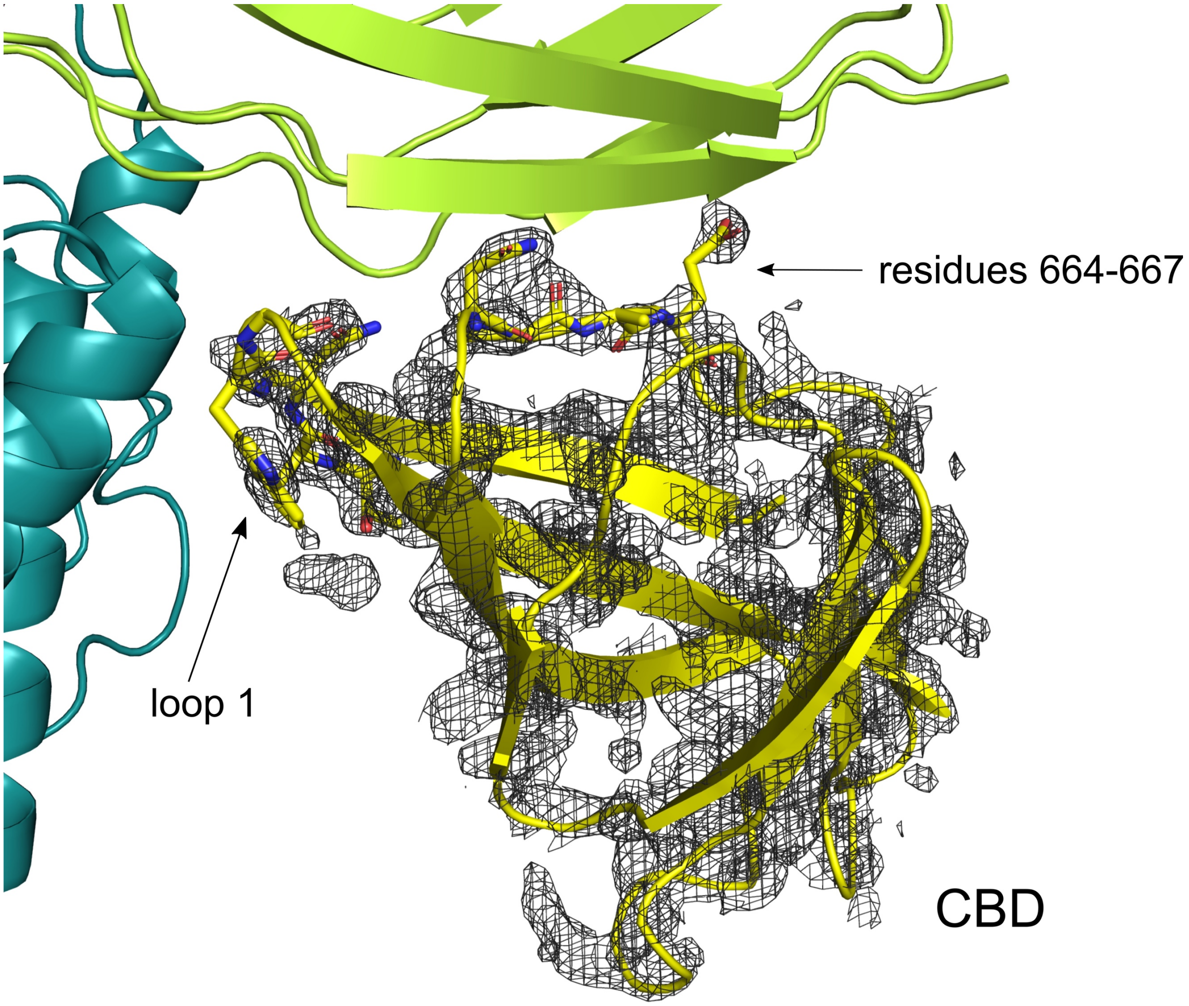

Figure S2. Close view of CBD contacts and omit map. Ribbon representation of ChiA74. CD, FnIII, and CBD are shown in dark cyan, green and lemon, respectively. Poor electron density was observed for CBD, probably due to the intrinsic flexibility of the domain and crystal packing. The mesh represents a 2mFo-DFc omit map calculated for the molecule B within the asymmetric unit, shown at a level of 2σ and carved at 1.6 Å around the domain. Residues in close contact are indicated as sticks. Most of the residues in CBD were modeled as alanines.

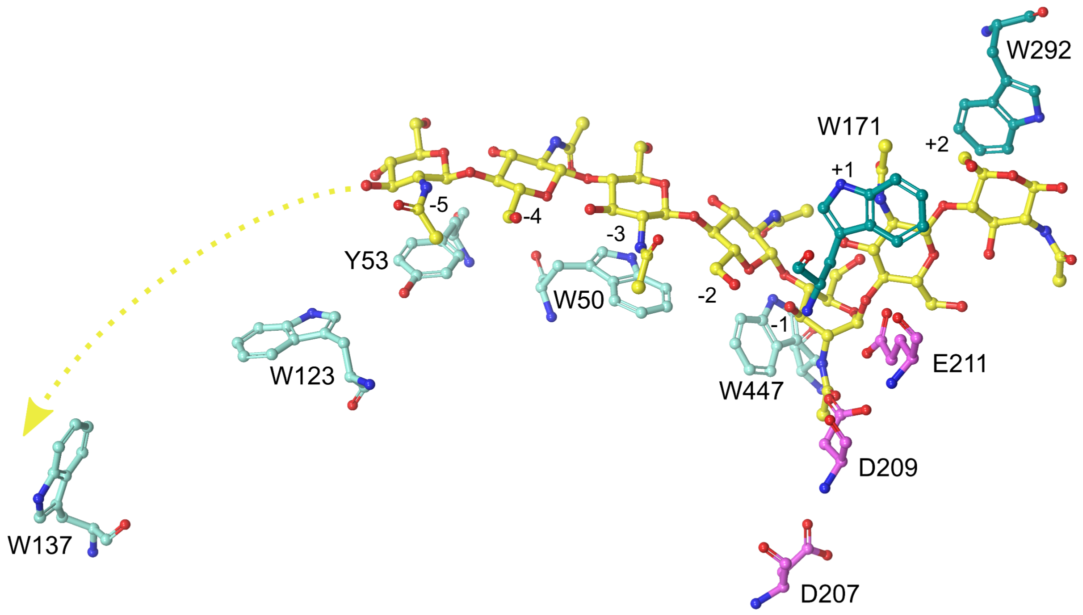

Figure S3. Substrate structural alignment with ChiA74. Ball and stick model of the substrate at the active cleft of ChiA74 is shown in yellow, subsites are numbered from -5 to +2, and correspond to the binding residues, light to dark cyan, respectively. Hydrolysis occurs at -1. The DXDXE motif is shown in magenta. ChiA74 may bind a longer substrate chain, dashed arrow. NAG 7 extracted from chitinase A *S. marcescens* (PDB 1EIB). Substrate superimposition and image rendering was done using Maestro program[36].

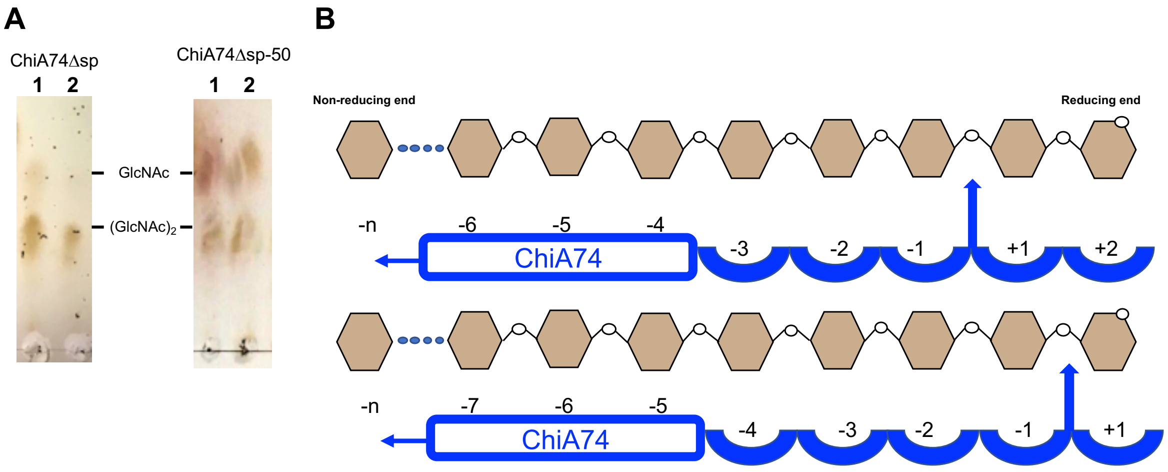

Figure S4. Figure S4. Thin-layer chromatography analysis of chitin-oligosaccharides produced by the hydrolytic action of ChiA74Δsp and the truncated enzyme version ChiA74Δsp-50. Panel A. Left and right, oligosaccharides produced from colloidal chitin (lane 1) and crystalline chitin (lane 2) treated with ChiA74Δsp and ChiA74Δsp-50. Molecular marker (Sigma-Aldrich, St. Louis MO, USA): β-(1,4) N-acetyl-d-glucosamine (GlcNAc) and chitobiose $(GlcNAc)_2$. Panel B. Schematic illustration of the putative processive activity of ChiA74Δsp and its truncated version ChiA74Δsp-50 on chitin. Subsites -1 and +1 of the enzyme are between the point of cleavage of the substrate. Subsites are numbered with increasingly negative numbers (-1, -2, -3, -4, -5…) away from the cleavage point toward the non-reducing end, whereas increasingly positive numbers (+1, +2, +3...) toward the reducing terminus. Reducing and non-reducing ends are shown at the right and left of the chitin. Dotted lines at the left, indicate that the polymer substrates are much longer to the non-reducing end than shown in the figure.

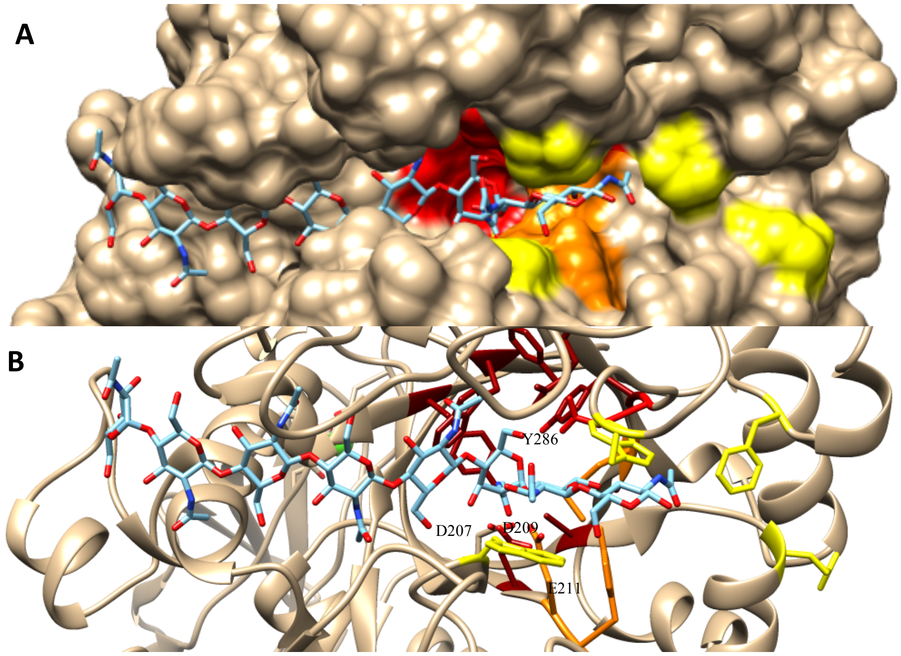

Figure S5. Putative catalytic mechanism of ChiA74 from *B. thuringiensis*. A) Prediction of interaction of Octa-N-acetyl-chitooctaose $(NAG)_8$ bound to the long semiclosed tunnel of the enzyme. The subsite -1, +1 and +2 are colored yellow, orange and red, respectively. Image was made by overlapping the chitinase of *S. marcescens* (PDB 1EIB). B) Active site region of ChiA74 showing the catalytic residues implicated the mechanism.
